# Can Digital Human Capital Promote Farmers’ Willingness to Engage in Green Production? Exploring the Role of Online Learning and Social Networks

**DOI:** 10.3390/bs15020227

**Published:** 2025-02-18

**Authors:** Siyu Gong, Ludi Jiang, Zhigang Yu

**Affiliations:** College of Economics and Management, Northeast Agricultural University, Harbin 150030, China; b210801012@neau.edu.cn (S.G.); s230801909@neau.edu.cn (L.J.)

**Keywords:** digital human capital, farmers’ willingness to engage in green production, online learning, social networks

## Abstract

The development of rural digitalization has become a key driving force for promoting green agricultural production. However, in practical operations, due to the insufficient digital skills and lack of necessary digital human capital among farmers, they struggle to distinguish between green production methods and traditional practices, which in turn reduces their willingness to adopt green production. This study employed empirical research methods to collect data from 854 farmers in China’s largest grain-producing region and used the Probit model to analyze the impact of digital human capital on farmers’ willingness to engage in green production. The results indicate that an increase in digital human capital can significantly enhance farmers’ willingness to engage in green production. Additionally, it was found that online learning can enhance farmers’ willingness towards green production, with informal online learning proving more effective. Further analysis revealed that social networks play a mediating role between digital human capital and farmers’ willingness to engage in green production. The study also explored the heterogeneous impact of digital human capital on different groups of farmers, highlighting that increases in digital human capital have a more pronounced effect on the willingness of small-scale farmers and middle-aged farmers to engage in green production. Therefore, continuously enhancing digital human capital, emphasizing diverse learning channels, and leveraging ’acquaintance networks’ to encourage farmers to improve their awareness of green production through digital platforms are critical for promoting sustainable green agriculture in developing countries.

## 1. Introduction

In the context of globalization, the United Nations has proposed a series of Sustainable Development Goals (SDGs) aimed at addressing numerous challenges faced by the world. Among these, sustainable agricultural development has become a widely recognized topic of importance. Sustainable agricultural development is the transformation of agricultural extensive management to fine management. It is not only conducive to the protection of the environment but also to the sustainable growth of food in China. China has promulgated many policies to promote the green development of agriculture, but there are still some obstacles to further promoting the green development of agriculture (K. M. Zhang & Wen, 2008). Although policy publicity, crop subsidies, and other measures have played an important role in the upgrading of green development in agricultural reclamation areas, these favorable conditions have not played a decisive role in the green production of farmers. On the one hand, it is difficult for farmers to adopt the green production mode directly because they usually lack the cultivated land area and supporting facilities equivalent to the reclaimed areas. On the other hand, farmers have deficiencies in human capital, and it is difficult for them to deeply understand the importance of green production from multiple dimensions, such as cost, income, and the environment. Considering that the current agricultural production and management in China is dominated by small farmers, if we cannot effectively stimulate small farmers to adopt green production behavior, the high-quality development of agriculture will be difficult to achieve. With the rapid advancement of information technology (Schreurs et al., 2017), the application of digital technologies has become a key factor in driving agricultural transformation, particularly in promoting efficient resource utilization and reducing environmental footprints (Granado-Díaz et al., 2024). However, in practice, despite the enormous potential of digital technologies, their application in agriculture, especially in developing countries, still faces numerous obstacles. One key issue is the dearth of digital human capital (Singh et al., 2023). Digital human capital refers to the information technology and computer skills acquired by individuals through education and training, which are essential for effectively utilizing digital tools (Bach et al., 2013). In the agricultural sector, enhancing farmers’ digital human capital not only aids them in better accessing and processing information required for agricultural production but also promotes the adoption and practice of green production behaviors (Kraay, 2019). For instance, research by Gbangou (2021) found that Weather and Climate Information Services (WCIS) can improve farmers’ ability to access digital information in West Africa, helping them cope with adverse weather conditions and make optimal decisions in agricultural production and sales. Digital human capital significantly influences farmers’ knowledge, management skills, perception, evaluation of production opportunities, and the integration of resources, thereby driving their production behaviors (Gong et al., 2024). Rich digital knowledge, digital skills, and heightened digital awareness enable farmers to promptly capture market information changes, perceive policy directions and market shifts, and make timely and accurate decisions, ensuring that their production activities align with demand (Syafuddin & Meidina, 2023; Sree & Rajender, 2023). Moreover, increasing digital human capital also strengthens the knowledge systems of farmers (Nwangwu et al., 2024), such as enabling those with higher levels of digital human capital to effectively manage crop production processes and maintain records related to agricultural safety (Khan et al., 2020). Additionally, some scholars argue that enhancing digital human capital helps farmers gain market information (Wang & Dong, 2023), increases their participation in e-commerce and digital financial activities (Magesa et al., 2020), and enhances engagement in online entertainment and community interactions (F. Li et al., 2023).

Although academia has conducted extensive research on digital human capital, there is currently limited study on how digital human capital specifically influences farmers’ willingness to engage in green production, particularly in the context of developing countries. Therefore, we have raised a forward-looking research question: can digital human capital improve farmers’ willingness to engage in green production? This study aims to fill this gap by examining the mechanisms through which digital human capital, in terms of online learning and social networking, influences farmers’ willingness to adopt green production methods. Given that many developing countries are undergoing rapid digital transformations, understanding this relationship is crucial for formulating effective policies to promote sustainable agricultural development.

Our research focuses on the interpretation of this issue. Compared to previous studies, the marginal contributions of this paper are as follows. Firstly, different from the previous focus on agricultural production, this paper is based on the background of the digital era. At the same time, this paper constructs a framework between digital human capital and farmers’ willingness to engage in green production, providing a new perspective on promoting green agriculture.

## 2. Literature Review

### 2.1. Digital Human Capital and Green Production Willingness

Farmers’ primary motivation for engaging in green production stems from the comparison of net returns between new and old production methods. Farmers will adopt a new production decision only if the net returns from adopting new technologies are greater than those from existing technologies (Schreurs et al., 2017). From the perspective of induced technological change theory, whether farmers adopt new production decisions depends on the scarcity of resources they possess or can access (M. Li et al., 2020). In other words, their production behavior is an endogenous response to shifts in critical economic variables. While the notion that green production by farmers is an environmentally and economically friendly approach has been widely accepted by most scholars (Koundouri et al., 2006; Kamara et al., 2023; Fu et al., 2009), this view is not entirely comprehensive. Yu et al. (2021) point out that factors influencing green production willingness, in addition to economic elements, include risk aversion during production. This provides a new perspective for research aimed at enhancing farmers’ willingness to engage in green production. Therefore, many scholars no longer focus their research on the economic incentives directed towards external motivations but instead choose to delve into the internalization of external incentives among farmers adopting green production technologies (Y. Zhu & Chen, 2024; Quan et al., 2024). In contrast to the rational decision-making driven by external economic incentives, farmers’ internal motivations are more sensitive and varied. For instance, when deciding whether to engage in green production, farmers place greater emphasis on the importance of green production and the ease of using green production technologies (Z. Zhu et al., 2024) rather than on economic incentives, which are typically anticipated benefits that are not immediately observable in production decisions. Therefore, the characteristics of green production are given greater weight by farmers. Furthermore, many scholars continue to explore the role of internal motivations. For example, Ozor et al. (2013) and Burnett et al. (2018) found that farmers’ farming experience, business scale, and livelihood capital significantly influence their willingness to engage in green production. Wheeler et al. (2019) and Luo et al. (2022) discovered that psychological factors, such as personal values and attitudes toward environmental protection, are also important determinants of farmers’ willingness to engage in green production. These studies reflect an essential problem, that the current level of human capital of farmers is insufficient, which will lead to missing the best opportunity of green production transformation.

Due to limited levels of human capital, farmers’ knowledge, judgment, and innovation ability may be constrained (Gulyaeva et al., 2023; Cordaro & Desdoigts, 2021), leading them to exhibit conservative thinking in agricultural production decisions, which keeps the level of production at a lower equilibrium (Hu et al., 2023). Consequently, enhancing farmers’ human capital can effectively promote their willingness to engage in green production. At present, effective strategies to improve human capital include government training (Ni et al., 2024) and neighborhood effects (Yamauchi, 2007), but these methods have certain limitations. For instance, government training is irregular and may be less effective for farmers with slower learning and absorption abilities. Neighborhood effects among farmers face challenges due to significant differences in individual skill levels, making it difficult to form effective momentum. To overcome these limitations and help farmers enhance their human capital, some scholars propose utilizing digital devices (F. Li et al., 2023). A key prerequisite for using digital devices is improving farmers’ digital human capital (Fabregas et al., 2019), requiring them to master basic digital knowledge, use digital devices for social interactions, apply digital technologies in various professional fields (Ma et al., 2022), and improve their digital financial, agricultural, and governance capabilities (Liu et al., 2024). On one hand, farmers with high digital human capital can efficiently integrate resources, transmit information, and communicate through digital platforms in a cost-effective and real-time manner (Gong et al., 2024; Malik, 2019). This informational advantage can reduce transaction costs and increase the returns from green production, motivating farmers to engage in green production (J. Zhang et al., 2024). For example, farmers can access vast amounts of green production knowledge through data searches, participate in online agricultural training via digital platforms, and reduce the cost of information search (Deichmann et al., 2016). They can also quickly capture market information through websites, further increasing their green production willingness (Manteaw et al., 2023). On the other hand, farmers with higher levels of digital human capital can leverage digital innovation to sell agricultural products and purchase agricultural inputs through e-commerce, gain insights into the green agricultural product market (G. Li & Zhang, 2024), and increase the added value of green agricultural products through green production (Zhong et al., 2022). Additionally, farmers with higher digital human capital have better opportunities to obtain loans (Yang et al., 2022) solve issues related to technology, funding, and time, and boost their willingness to engage in green production.

**Hypothesis** **1.** *Digital human capital has a positive impact on farmers’ willingness to engage in green production.*

### 2.2. Mediating Role of Online Learning

Recent studies have confirmed that the study of production knowledge organized by the government, the guidance of experts in agricultural extension, and the communication between peers can effectively improve the quality of agricultural production (Glendenning et al., 2010; Takahashi et al., 2019). Online learning has gradually become common with the development of the times (Singh & Thurman, 2019). According to the theory of digital human capital, the advantage of online learning lies in that it uses internet technology to meet the needs of farmers for personalized learning, realizes independent learning, breaks the time and place restrictions for organizing offline learning, reduces the difficulty and cost of organizational learning, and expands training coverage (Castiblanco Jimenez et al., 2020). At the same time, farmers with higher digital human capital level can save and share the training content in the form of videos or electronic documents, which is conducive to their repeated learning of difficult knowledge and the exchange and sharing of agricultural technology knowledge (Abdon & Raab, 2005). Online learning has significant advantages in policy popularization, theoretical training, and technical training with little difficulty and production introduction, as well as systematic training that covers a large amount of content over a longer duration. At present, online learning can be divided into two main types, namely formal and informal online learning (Czerkawski, 2016; Schwier, 2012). Formal online learning is an online learning method led by the government, scientific research institutes, and village collectives. Through the official website for internet training, farmers can learn green production knowledge and green production skills by themselves. Moreover, farmers can choose experts in different fields and agricultural extension workers according to their own preferences. Formal online training has the advantages of more professional image and video content. Using pictures and text forms to show farmers a variety of green production methods, green production process, and technology use can realize the visualization and accessibility of green production in a more professional way (Doruchowski et al., 2013). Informal online learning is an online learning for farmers to learn independently on the third-party platform. In this mode, farmers can learn the relevant knowledge of green production and green production skills with other large-scale growers through TikTok and other apps. Large-scale farmers use live broadcast to interact with farmers who need knowledge acquisition in real time. Farmers are more receptive to the knowledge and methods taught by large-scale farmers who are also growers. The third-party APP has the advantage of low threshold, which can stimulate farmers with a high level of digital human capital to disseminate videos and pictures related to green production (Deng et al., 2022). Farmers can apply the skills and methods learned from online training to solve the problems in the production process (Czerkawski, 2016; Narine et al., 2019). These include, for example, methods for diluting pesticides to improve their utilization rate; policies and regulations related to the green application of pesticides and fertilizers; and methods and standards for returning straw to the field. Farmers can not only improve their environmental awareness and environmental awareness (Despotović et al., 2021) but also independently distinguish the types of pesticides and grasp the quality, application amount, application method, utilization rate, and application effect of pesticides; therefore, the possibility of adopting green production technology is greater.

**Hypothesis** **2.** *Digital human capital can increase farmers’ willingness to engage in green production by promoting their online learning.*

**Hypothesis** **2a.** *Digital human capital can increase farmers’ willingness to engage in green production by promoting formal online learning.*

**Hypothesis** **2b.** *Digital human capital can increase farmers’ willingness to engage in green production by promoting informal online learning.*

### 2.3. Moderating Role of Social Network

Social network refers to a relatively stable relationship system formed by interaction between individual members of society, which focuses on the interaction and connection between people. Social interaction will affect people’s social behavior (Fincher et al., 2011). Social network representing various social relations is a kind of social structure composed of many nodes that usually refer to individuals. From casual acquaintances to people with close relationships, they are connected in series through these social relations. The social network of farmers refers to the connection with family members, friends, and acquaintances, which is a strong network relationship (Skaalsveen et al., 2020). First, with the increasing use of social networks, the deeper the embedding among network members, the deeper the impact on individuals (Yan et al., 2025). Farmers with high digital human capital will indirectly drive the people around them to improve their digital capital. Second, social networks can not only form extensive connections among farmers but also have strong trust and familiarity among their members (Agnihotri et al., 2022), which easily forms a sort of snowball effect in reputation effect. The stronger this social network is, the higher the degree of mutual familiarity among its members, which leads to a lower probability of opportunistic behaviors and facilitates the formation of emotional trust among network members (Sherchan et al., 2013). This is conducive to the protection of knowledge sharing and transfer (Dilleen et al., 2023; Fisher et al., 2018), enabling members to share knowledge related to green production selflessly. Under such circumstances, members can exchange knowledge related to green production more conveniently, promoting the dissemination of green production knowledge among farmers (Sah et al., 2021). Ultimately, as interactions and exchanges deepen between farmers and members of their personal networks, their similarities increase, leading to similar understandings and perceptions of green production (Kiptot & Franzel, 2019). Farmers become more willing to provide information and advice to each other, and the content of tacit knowledge related to green production increases during these exchanges. The improved speed and quality of knowledge transfer encourage farmers to enhance their digital capabilities, fostering deeper learning and communication mechanisms among themselves. This stimulates digital creativity and innovation, increases innovative outputs, and raises the willingness of farmers to engage in green production.

**Hypothesis** **3.** *Social networks play a moderating role between digital human capital and farmers’ willingness to engage in green production.*

## 3. Methodologies and Data

### 3.1. Sample Selection

The data utilized in this study were gathered by the investigation team of “Farmers’ Green Agricultural Production” from Northeast Agricultural University. The team conducted a random sampling of farmers engaged in the cultivation of rice, corn, and soybeans in Heilongjiang Province. Two primary factors led to the selection of Heilongjiang Province as the research site: on the one hand, it is China’s largest grain-producing region and one of the world’s four largest black soil areas. On the other hand, Heilongjiang Province is dedicated to advancing the collaborative digital and green transformation and development within its borders. Based on economic development status and transportation accessibility, 1 to 3 townships were selected from each county, and then 15 to 30 farmers were randomly chosen to participate in the survey.

The questionnaire was structured into three sections. The initial section sought demographic information from the participants. The second section aimed to assess the respondents’ digital human capital, encompassing digital basic capability (DBC), digital communication capability (DCC), digital content creation capability (DCCC), and digital financial participation capability (DFPC). The final section included inquiries regarding the respondents’ willingness to engage in green production. In total, 873 surveys were administered, resulting in 854 valid responses post-screening. Overall, the sample distribution within the specified region of China (Heilongjiang Province) mirrors the broader national demographic profile.

### 3.2. Variables Definitions

#### 3.2.1. Explained Variable

The explained variable is the farmers’ green production willingness, which is a binary discrete variable. If farmers are willing to carry out green production, the value is 1, otherwise, the value is 0.

#### 3.2.2. Explanatory Variable

Digital human capital refers to the ability to recognize, understand, and utilize digital information and resources as the basic skills of the digital era (Ivanova et al., 2020). Digital human capital can help farmers quickly adapt to the digital era and enhance their personal capabilities to adapt to the changing new environment (Lin et al., 2023). Digital human capital is the ability of farmers to search for information, connect with others, transform and create digital information, and use digital financial means to meet their production needs through apps, the internet, and other digital devices or tools (Kanter et al., 2023; Nogueira et al., 2022). The explanatory variable of this study is digital human capital, which can affect the farmers’ willingness to engage in green production. Drawing on the Digital Competence Framework 2.2 released by the European Union, it is divided into four dimensions, including digital basic capability (DBC), digital communication capability (DCC), digital content creation capability (DCCC), and digital financial participation capability (DFPC) (see Appendix A). Among them, digital basic capability refers to the farmers’ ability to operate digital equipment and acquire digital information. Digital communication capability refers to the ability of farmers to communicate, share, participate, and express themselves when using digital equipment. Digital content creation capability refers to the ability of farmers to integrate and reuse digital content in the digital environment, as well as the understanding of digital constraints and permits. Digital financial participation capability refers to the digital consumption and investment ability of farmers.

The assessment of digital human capital among farmers in the sample area reveals that the largest group of farmers falls within digital human capital score range of 2.5 to 3, accounting for 35.13% of the sample. Farmers with digital human capital scores between 2 and 2.5 and between 3 and 3.5 constitute the next largest groups, representing 31.97% and 18.85%, respectively. Only 6.56% of farmers have higher digital human capital levels. This indicates that, overall, farmers’ digital human capital is relatively low, with very few farmers possessing high levels of digital human capital. However, farmers’ ability to communicate with the skilled use and daily use of digital equipment is constantly improving. These findings align with the conclusions of the “Survey Analysis Report on Digital Literacy in Rural China under the Background of the Rural Revitalization Strategy”, published by the Information Research Center of the Chinese Academy of Social Sciences. The report highlights that the level of digital human capital in rural areas is lower than in urban areas. There is a clear need to further enhance the digital capabilities of rural populations, strengthen farmers’ digital skills and literacy, and promote high-quality development in agriculture and rural areas.

#### 3.2.3. Other Variables

This paper mainly explores the mechanism role of online learning between digital Human Capital and farmers’ willingness to engage in green production, as well as the moderating role of social network. Online learning is divided into formal online learning and informal online learning. Formal online learning focuses on government-led training programs, scientific research institutions, and village collective. These programs are typically delivered through official websites and mobile applications designed specifically for agricultural education. Key platforms include the Ministry of Agriculture’s official website, local agricultural extension services’ websites, and specialized apps like “Yunshang Smart Farmer”. The content provided by these platforms includes structured courses on green production techniques, policy updates, technical training videos, and interactive sessions with agricultural experts. In contrast, informal online learning primarily occurs through third-party platforms such as TikTok, WeChat, and other social media channels. Farmers use these platforms to access a variety of content related to green production, including short instructional videos, live streams from experienced farmers, and community forums for discussing practical experiences. For example, large-scale growers often share their knowledge and best practices through live broadcasts on TikTok, while WeChat provides detailed articles and multimedia content on green production methods and market trends. Referring to the research ideas of Peeters et al. (2014), Dabbagh and Kitsantas (2012), and Šūmane et al. (2018), formal online learning is measured by asking farmers to evaluate three aspects, namely ”online agricultural production knowledge learning frequency organized by the village”, “online training frequency of agricultural technology extension personnel”, and “the use of relevant APP about agricultural production recommended by the village leaders”. In addition, informal online learning is also measured by giving farmers three tasks in turn. These include “learn the relevant content of agricultural green production independently through short video platform”, “discuss the experience of agricultural green production with non-professionals online” and “use the official account of agricultural WeChat (or on the webpage) to solve the latest agricultural policies and green production information”. We used the Likert scale for assigning values to the degree of formal online learning or informal online learning in this paper. If farmers have not engaged in online learning, the value is assigned as 1. If they have, they are assigned values from 2 to 5, reflecting the frequency of their study. These values are as follows: “Never = 1, Seldom = 2, Occasionally = 3, Sometimes = 4, Often = 5”. The entropy value method is used to calculate the weight of each index, as shown in Table 1. Moreover, in the survey, we found that farmers prefer platforms that offer easy-to-understand and practically applicable content. Specifically, they value content that addresses immediate concerns, such as pest management techniques, soil health improvement methods, and market information for green products. Informal platforms like TikTok and WeChat were particularly favored for their ability to provide timely and context-specific information.

According to social network theory, this article has constructed comprehensive indicators for social networks from three dimensions of network breadth, connection strength, and network’s upward sociability. Network breadth uses human contact expenditure as a proxy variable, with the “the total income and expenditure of human relations (ten thousand yuan)” as the indicator. Connection strength is measured by the item “frequency of interaction with relatives, friends and neighbors”. Network’s upward sociability is measured by the “frequency of communication with village leaders (and public officials above the village level)”. A 5-pointLikert scale is used for scoring, and farmers should choose a value from 1 to 5 based on their actual situation. These values are as follows: “Never = 1, Seldom = 2, Occasionally = 3, Sometimes = 4, Often = 5”. The weight of the arithmetic mean is measured in Table 2.

#### 3.2.4. Control Variable

Referring to the research by Ozor et al. (2013) and M. Li et al. (2020), the control variables selected in this article include farmer characteristics (gender, age, education level, political status, part-time employment status), operational characteristics (years of agricultural production, number of agricultural laborers, participation in cooperatives, area of grain cultivation, farming income, farmland quality, agricultural insurance), natural characteristics (natural disasters), and village characteristics (distance to town).

Table 3 provides the descriptive statistics for this article.

### 3.3. Model Set-Up

#### 3.3.1. Probit Model

Farmers’ willingness to engage in green production is a binary variable. If the farmer is willing to conduct green production, the value is assigned as 1, otherwise, the value is assigned as 0. Therefore, this paper selects the Probit model with good explanatory power of binary variables to analyze the influence of digital human capital on farmers’ willingness to engage in green production.

(1) $$P({Willingness}_{ij}=1|{Digital}_{ij})=\Phi (\rho_{0}+\rho_{1}{Digital}_{ij}+\rho_{2}{Control}_{ij}+\sigma_{j}+\varepsilon_{ij})$$

In Equation (1), Φ(∗) represents the cumulative distribution function, ${Willingness}_{ij}$ represents farmers’ willingness to engage in green production, ${Digital}_{ij}$ represents digital human capital of farmers, ${Control}_{ij}$ is a series of control variables affecting farmers’ willingness to engage in green production, $\rho_{0}$ is a constant term, $\rho_{1}$ and $\rho_{2}$ are the coefficients to be estimated, $\sigma_{j}$ is city-level regional effect, and $\varepsilon_{ij}$ is error term.

#### 3.3.2. Mediation Effect

This paper explores the possible mediating effects based on Equation (1) by further identifying the causal relationship of the core explanatory variables on the mediating variables. The model is as follows:

(2) $${Willingness}_{ij}=\alpha_{0}+\beta_{0}{Digital}_{ij}+c_{0}{Control}_{ij}+\sigma_{j}+\mu_{ij}$$

(3) $${Online}_{ij}=\alpha_{1}+\beta_{1}{Digital}_{ij}+c_{1}{Control}_{ij}+\sigma_{j}+\mu_{ij}^{\prime }$$

(4) $${Willingness}_{ij}=\alpha_{2}+\beta_{2}{Digital}_{ij}+c_{2}{Control}_{ij}+d_{2}{Online}_{ij}+\sigma_{j}+\mu_{ij}^{\prime \prime }.$$

In Equations (2)–(4), *Online_i_* is online learning (divided into formal online learning and informal online learning); *α*_0_, *α*_1_, *α*_2_ are constant terms; *β*_0_, *β*_1_, *β*_2_, *c*_0_, *c*_1_, *c*_2_, *d*_2_ are the coefficients to be estimated; and *μ_ij_*, $\mu_{ij}^{\prime }$, $\mu_{ij}^{\prime \prime }$ are error terms.

#### 3.3.3. Moderation Effect

When both the core explanatory and moderator variables are continuous variables, the moderating effect should be tested in the form of an interaction term. In order to further explore the moderating effect of social networks between digital human capital and online learning, we added the interaction term of digital human capital and social networks in the regression model to investigate the moderating effect of social networks, and constructed the following models:

(5) $${Online}_{ij}=\alpha_{3}+\beta_{3}{Digital}_{ij}+c_{3}{Soc}_{ij}+d_{3}{Digital}_{ij}*{Soc}_{ij}+e_{3}{Control}_{ij}+\sigma_{j}+\mu_{ij}^{\prime \prime \prime }.$$

In Equation (5), ${Online}_{ij}$ and ${Digital}_{ij}$ are online learning (divided into formal online learning and informal online learning); ${Soc}_{ij}$ is the moderator variable (social network); ${Digital}_{ij}*{Soc}_{ij}$ are interaction terms between the core explanatory variables and the moderator variable (interaction term between digital human capital and social network); $\alpha_{3}$ is a constant term; $\beta_{3},c_{3},d_{3},e_{3}$ are the coefficients to be estimated; and $\mu_{ij}^{\prime \prime \prime }$ is error term. According to the symbol $d_{3}$, the moderating effect of social networks is determined.

## 4. Empirical Results and Discussion

### 4.1. Baseline Results

We used the binary Probit model to analyze the influence of DHC on farmers’ willingness to engage in green production (Table 4). As shown in Table 4, Column (1) is the estimated result of the impact of DHC on farmers’ willingness to engage in green production. Column (2) is the marginal effect result of human capital on farmers’ GPW. The results showed that DHC has a significant effect on farmers’ willingness to engage in green production, with an influence coefficient of 0.3862. The statistical significance level is 1%, indicating that the continuous improvement of farmers’ DHC has promoted the continuous improvement of farmers’ GPW. In terms of the marginal effect results, farmers increased DHC by one unit, and their GPW increased by 0.1185. Column (3) is the estimated result of the impact of different dimensions of DHC on farmers’ willingness to engage in green production. Column (4) is the result of the marginal effect of different dimensions of DHC on farmers’ GPW. The results show that digital basic capability (DBC), digital communication capability (DCC), and digital financial participation capability (DFPC) all have a significant impact on farmers’ GPW. The influence coefficient were 0.1241, 0.1165, and 0.0998, respectively. Furthermore, the statistical significances were at the 1%, 5%, and 5% statistical levels, indicating that with DBC, DCC, and DFPC, the continuous improvement of farmers’ GPW is promoted. From the perspective of marginal effects, each one-unit increase in DBC enhances farmers’ willingness to engage in green production by 0.0379. Each one-unit increase in DCC enhances their willingness by 0.0356. Each one-unit increase in DFPC enhances their willingness by 0.0305. Hypothesis 1 was verified.

From the control variables, EDU, AGRI, LABOR, and LAND have a significant impact on their GPW, indicating that the higher the education level of farmers, the longer the years engaged in agricultural production, the fewer the number of agricultural laborers, and the better the quality of the cultivated land managed, the stronger the GPW of farmers.

### 4.2. Endogenicity Test

DHC may have endogenous problems with farmers’ GPW because of the two-way causal relationship. Theoretically, the model may contain some missing variables that are difficult to effectively control, and which may have simultaneous effects on DHC and GPW. In order to improve the reliability and accuracy of the benchmark regression results, we used the instrumental variable method to test endogeneity. This article selected “the average level of DHC of other farmers in the same village” as the instrumental variable of DHC.

The results of the regression using IV-2SLS are shown in Table 5. In the first stage, the average level of the influence of the DHC of other farmers in the same village on the personal DHC of farmers was significantly positive at the statistical level of 1%, and the influence coefficient was 0.3934. In the second stage, the influence of DHC on GPW was significant at the statistical level of 1%, with an influence coefficient of 0.4557. The F-statistic is greater than 10, which exceeds the cutoff value of 16.380 for the Stock–Yogo weak instrumental variable test, rejecting the null hypothesis of weak instrumental variables. This indicates that the model does not have a weak instrumental variable problem. In addition, the model is considered adequate confirmation, as the number of instrumental variables used in this paper is the same as the number of endogenous variables. In conclusion, the instrumental variables selected in this study were valid. Moreover, after alleviating the endogenous problem, the improvement of DHC still helps to improve farmers’ GPW.

### 4.3. Robustness Test

In this study, three methods were used to verify the robustness of DHC on farmers’ GPW. First, we used the Logit model instead of the Probit model to reverify the impact of DHC on farmers’ GPW. As shown in Table 6 Columns (1)–(2), the influence direction and significant results of DHC on farmers’ green production intention have strong consistency, and the effect results are significant at the statistical level of 1%. Second, the calculation method of changing the core explanatory variables was adopted to check the robustness of the regression results. After re-measuring the DHC level of farmers with the weighted average of DBC, DCC, DCCC, and DFPC, the relationship between DHC and farmers’ GPW was investigated. The results are shown in Table 6 Columns (3)–(4). DHC had a positive impact on farmers’ willingness to make green production, and the influence coefficient was 0.3589, which indicated that after changing the measurement method of DHC, there was still a positive and significant relationship between the main variables. The empirical results have strong robustness. Third, this article reduced 1% and 99% of the samples to verify the impact of DHC on farmers’ GPW. According to Table 6 Column (5)–(6), DHC has a significant positive impact on farmers’ GPW, the effect results are significant at the statistical level of 1%, and the robustness test results are strongly consistent with the benchmark regression results. The above three methods show that the baseline results are relatively robust.

### 4.4. Results of the Mediating

This article used the stepwise regression to test the mediating role of online learning in the relationship between DHC and farmers’ GPW. As shown in Table 7 Column (1), the influence of DHC and farmers’ GPW passed the significance test at the 1%. The influence coefficient was 0.1229, indicating that DHC had a significant positive influence on farmers’ GPW. As shown in Table 7 Column (2), the impact of formal online learning (FOL) on farmers’ GPW passed the significance test at the 1% level. The influence coefficient was 0.0529, indicating that FOL has a significant positive impact on farmers’ GPW. According to the results of Table 7 Column (3), the influence of informal online learning (IOL) on farmers’ GPW passes the significance test at the 1% level. The influence coefficient is 0.0698, indicating that IOL has a significant positive impact on farmers’ GPW. As shown in Column (4), DHC still has a positive impact on farmers’ GPW after leading into FOL variables; it passed the significance test at the 1% level, and the influence coefficient was 0.0994. Compared with Column (1), the coefficient of DHC in Column (4) decreased (0.0994 < 0.1229). It means that FOL played a partial intermediary role in DHC affecting farmers’ GPW. As shown in Column (5), DHC still has a positive impact on farmers’ GPW after leading into IOL variables; it passed the significance test at the 1% level, and the influence coefficient was 0.0894. Compared with Column (1), the coefficient of DHC in Column (5) decreased (0.0894 < 0.1229), which means that IOL played a partial intermediary role in the impact of DHC on farmers’ willingness to engage in green production. As shown in Column (6), after drawing into the variables of formal or IOL, the impact of DHC on farmers’ GPW passed the significance test at the 1% level, and the influence coefficient was 0.0757. Compared to Column (1), the coefficient of DHC in Column (6) decreased (0.0757 < 0.1229), indicating that the addition of FOL and IOL also played an intermediary role. In addition, after FOL and IOL were added, the two coefficients were 0.0256 and 0.0340, respectively, which passed the significance tests of 10% and 5%, respectively. Therefore, the results found that DHC not only directly affects farmers’ willingness to engage in green production, but also improves farmers’ GPW through FOL and IOL. DHC has improved farmers’ willingness to produce green through online learning, and the effect of informal learning is better than FOL. Hypothesis 2 was initially verified.

### 4.5. Results of the Moderating

In order to test the moderating role of social networks in DHC and online learning, as well as the moderating role of mediation in DHC and farmers’ GPW, this article used SPSS software to centralize the data and analyze the boundary role of social networks (SN). As shown in Table 8 Column (1), the influence coefficient of DHC on FOL was 0.3938, and the significance test was at the 1% level. DHC has a significant promotion effect on FOL. Column (2) is the regression results after the addition of the moderating variable SN, indicating that the influence coefficient of DHC on FOL was 0.3945, which passed the significance test at the 1% level. As shown in Column (3), the influence coefficient of the interaction term of DHC and SN on FOL was 0.0550, which passed the significance test at the 10% level, and the effect of DHC on FOL passed the significance test at the 1% level, indicating that the SN positively moderated the relationship between DHC and FOL. As shown in Column (4), the influence coefficient of DHC on IOL was 0.7261, and the significance test was at the 1% level. DHC has a significant promotion effect on IOL. Column (5) is the regression results after the addition of the moderating variable SN, indicating that the influence coefficient of DHC on IOL was 0.7270, which passed the significance test at the 1% level. Column (6) is the result of adding the interaction term of the moderating variable SN, indicating the interaction terms between DHC and SN. The influence coefficient of the interaction term of DHC and SN on IOL was 0.1023, and the significance test was at the 1% level. The influence of DHC on IOL and the influence of SN on IOL all passed the significance test at the 1% level, indicating that SN positively moderate the relationship between DHC and IOL. According to the results, SN had a moderating effect on DHC, FOL, and IOL. The results show that social networks significantly expand the effect of online learning by providing timely support. Farmers who build better social networks are more engaged in online learning content, have faster access to practical advice, and have a stronger sense of community support. This enhances their overall learning experience and motivation for green production. For example, farmers discuss pest management techniques in their daily life and receive real-time feedback, thus improving their application of these technologies. These interactions not only enrich the retention of knowledge but also facilitate continuous learning and practice improvement. Therefore, Hypothesis 3 was initially verified.

**Figure 1:**
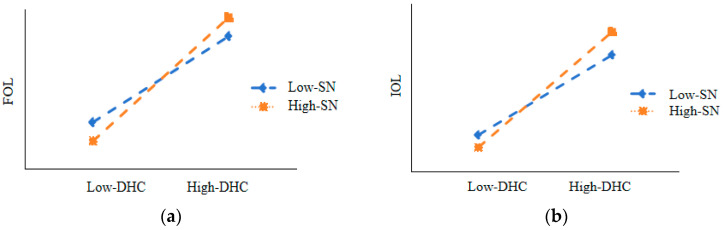
The moderating role of social networks (SN) between digital human capital (DHC) and online learning (ONL). (**a**) The moderating role of SN between DHC and formal online learning (FOL). (**b**) The moderating role of SN between DHC and informal online learning (IOL).

Hypothesis 3 proposed a moderated mediation model. This subsection further tested this hypothesis by using the unbiased confidence intervals of the conditional indirect effects estimated by the Monte Carlo simulation method. As shown in Table 9, Figure 1a,b, we performed 20,000 Bootstrap sampling tests. In the FOL, High-SN (β = 0.46650, SE = 0.0654, 95% non-biased confidence interval [0.3384,0.5946], excluding 0) had a stronger moderating effect than Low-SN (β = 0.3238, SE = 0.0648, 95% unbiased confidence interval [0.1968, 0.4509], excluding 0) between DHC and FOL. In the IOL, High-SN (β = 0.8610, SE = 0.0757,95% non-biased confidence interval [0.7127, 0.1.0093, excluding 0) had a stronger moderating effect than Low-SN (β = 0.5956, SE = 0.0750, 95% unbiased confidence interval [0.4486, 0.7427], excluding 0) between DHC and IOL. The above results indicate that the SN plays a moderated mediating role, meaning that SN indirectly moderates the relationship between DHC and online learning.

### 4.6. Analysis of Heterogeneity

In order to further analyze the influence of DHC on farmers’ GPW, this paper discusses the heterogeneity of DHC on farmers’ GPW from the perspectives of farmers’ age and farmland scale.

#### 4.6.1. Heterogeneity of Age

Using the World Health Organization (WTO)’s criteria for young, middle-aged, and elderly individuals, the sample farmers were divided into a young, a middle-aged, and an elderly group by combining the agricultural production situation in China. Farmers aged between 45 and 60 (including 45 and 60) were sorted into the middle-aged group. Farmers over 60 years were sorted into the elderly group. As shown in Table 10 Columns (1)–(2), DHC had a significant positive impact on the GPW of young farmers, and the influence coefficient was 0.3046, which was significant at the statistical level of 1%. In other words, DHC can significantly increase the willingness of young farmers to produce green products. As far as the marginal effect is concerned, the young farmers’ willingness to carry out green production increased by 0.0926 for every unit of DHC. According to Column (3)–(4), DHC had a significant positive impact on the GPW of middle-aged farmers, and the influence coefficient was 0.5602, which was significant at the statistical level of 1%. It means that DHC significantly improved the GPW of middle-aged farmers. As far as the marginal effect is concerned, the middle-aged farmers’ willingness to engage in green production was increased by 0.1135 for every unit of their DHC level. According to the above results, we can find that, compared with young farmers, DHC has a stronger effect on promoting the GPW of middle-aged farmers. As shown in Table 10 Column (5)–(6), DHC had a significant positive impact on the GPW of elderly farmers, and the influence coefficient was 0.4768, which was significant at the statistical level of 1%. It means that DHC significantly improved the GPW of elderly farmers. As far as the marginal effect is concerned, the elderly farmers’ willingness to engage in green production increased by 0.1097 for every unit of their DHC level. A possible reason for this is that although elderly farmers have more experience and deeper understanding of agricultural production, they tend to prefer to use traditional methods for production (White, 2012). With the continuous development of digital technology, there are new possibilities for the green transformation of agricultural production. Middle-aged and elderly farmers have better DHC and master certain digital skills, which can help them better understand the importance and advantages of green production, enabling them to engage in it more efficiently. Young farmers have innate advantages in digital application, and, relatively speaking, they have a better awareness of environmental protection (Irungu et al., 2015). In this scenario, DHC has a stronger effect on promoting the GPW of middle-aged farmers (aged 45 to 60).

#### 4.6.2. Heterogeneity of Farmland Scale

Farmers were divided into two groups, namely small-scale farmers and large-scale farmers, according to the median cultivated land area. Then, we explored the heterogeneity of the influence of DHC on the GPW of farmers with different planting scales (Table 11). As shown in Column (1)–(2), the positive effect of DHC on the GPW of small-scale farmers was significant, with an influence coefficient of 0.4560, which was significant at the 1% statistical level. In other words, DHC can significantly increase the willingness of small-scale farmers to produce green products. As far as the marginal effect is concerned, the small-scale farmers’ willingness to carry out green production will be increased by 0.1382 for every unit of DHC. As shown in Column (3)–(4), the positive effect of DHC on the GPW of large-scale households was significant, with an influence coefficient of 0.3378, which was significant at the 1% statistical level. This also means that DHC significantly improved the willingness of large-scale households to engage in green production. In terms of marginal effect, the large-scale farmers’ willingness to carry out green production increased by 0.1017 for every unit of DHC. According to the above results, compared with large-scale households, DHC has a stronger effect on promoting the GPW of small-scale farmers. A possible reason for this is that small-scale farmers often face challenges such as limited resources and asymmetric information, and DHC can help them to better obtain and use information related to agricultural production. Production information barriers can be broken by small-scale farmers through the help of digital information and networks. Furthermore, information about green production methods, market trends, and environmental policies can be more easily accessed. As DHC can comprehensively enhance personal environmental awareness and enhance the willingness of agricultural green production, it has a stronger effect on promoting the GPW of small-scale farmers.

## 5. Discussion

The main purpose of this study was to examine the impact of digital human capital on farmers’ green production willingness and the pathways between them, based on the digital human capital theory. Our research findings show that digital Human Capital will have a positive impact on farmers’ green production willingness through online learning. In addition, this study also examines the boundary conditions of social networks in the relationship between digital Human Capital and farmers’ willingness to engage in green production.

### 5.1. Theoretical Implications

Firstly, while previous studies have highlighted the importance of enhancing human capital for decision-making changes (Schmidt & Heidenreich, 2018; Jansen et al., 2013), existing research on the relationship between digital human capital and farmers’ willingness to engage in green production lacks a logically unified analytical framework. This study integrates digital human capital theory and competence theory to construct a research framework that examines the interplay between digital human capital and farmers’ willingness to engage in green production. Unlike previous studies, this research expands the scope of digital human capital theory. Digital human capital reflects the extent to which farmers value digital content and their ability to acquire, utilize, and transform it. Enhancements in digital human capital not only improve farmers’ ability to obtain, share, use, and process information related to green agricultural production but also enhance their capacity to create, iterate, and disseminate information.

Secondly, this study proposes that improvements in digital human capital drive the mechanisms of formal and informal online learning and reveals the critical role of online learning in the accumulation of green production knowledge among farmers. This addresses the disparities in farmers’ willingness to engage in green production due to information asymmetry, providing scholars with a perspective on supplementing the mechanism research between digital human capital and farmers’ green production behaviors through the lens of online learning.

Finally, this study examines the moderating effect of social networks on the relationship between digital human capital and farmers’ willingness to engage in green production, broadening their theoretical boundaries. The study also finds that the expansion of social networks facilitates farmers’ understanding of green production through online learning, enhancing their willingness to engage in green production.

### 5.2. Practical Implications

This study provides significant implications for policymakers and agricultural producers. Firstly, to address the challenge of low initial levels of digital human capital among smallholder farmers, particularly for disadvantaged groups, governments should prioritize the development of tailored training programs focusing on practical applications such as using agricultural apps and platforms. These programs should include structured courses and hands-on workshops, offered through community learning centers and partnerships with universities or agricultural extension services. Governments must ensure access to affordable devices and reliable internet connectivity by providing subsidies or financing options. Additionally, policymakers should introduce incentive programs, such as financial subsidies for technology adoption and tax breaks for participating in digital human capital programs. To support disadvantaged farmers specifically, governments should provide specialized training and additional resources, ensuring inclusiveness and effectiveness. Operational details include setting up mobile training units to reach remote areas, creating localized content in local resources, and establishing peer mentoring networks to provide continuous support. While improving the digital human capital of farmers, it will enhance their willingness and ability to carry out green production.

Secondly, it is important to use different ways to help farmers learn about green production. Online learning is very useful for gaining new knowledge and encouraging farmers to be more involved in online platforms. While improving formal online training by agricultural experts, we should also use the influence of internet personalities and successful large-scale farmers to boost farmers’ interest in green production. Using a mix of learning methods like videos, mobile apps, and online platforms can make learning easier and more interesting for farmers. For example, working with popular internet figures who are well-known in rural areas can help spread information more effectively. Similarly, involving successful large-scale farmers as extension agents can provide practical advice and inspire confidence in trying new farming practices. These large-scale farmers can share their success stories, showing how green production methods can bring real benefits. Additionally, creating opportunities for farmers to learn from each other through social networks can build a supportive community, increasing motivation and commitment to green production. By combining expert-led online training with the influence of large-scale farmers and using various learning methods, we can greatly improve farmers’ digital skills and their willingness to adopt green production techniques.

Lastly, the “social acquaintance network”, which includes everyday interactions between neighbors and community members, is a vital channel for accessing production information and requires further activation. To build stronger social networks, communities can organize regular meetups and workshops where farmers can share their experiences and learn from each other. For example, local village committees can facilitate small group discussions to keep everyone connected. Such social networks allow farmers to ask questions, seek advice, and share success stories, fostering an environment that encourages continuous learning. Additionally, involving local leaders and influencers who are trusted within the community can help promote the construction of social networks. By strengthening these everyday social networks, we can significantly improve farmers’ digital human capital and their ability to benefit from online learning. This holistic approach not only enhances individual skills but also creates a collective effort towards sustainable agricultural practices. This will drive the green transformation of agricultural production by ensuring that farmers have both the knowledge and the support they need to enhance their willingness to engage in green production.

### 5.3. Further Research and Limitations

Despite our study’s attempt to enrich the existing literature by investigating the role of digital human capital in farmers’ willingness to engage in green production, several limitations are acknowledged that necessitate further research. First, while our sample size was adequate, it may not fully represent the broader agricultural population due to regional variations. Specifically, the Heilongjiang region, where our study was conducted, has unique characteristics compared to other agricultural regions. These include its climate, soil conditions, and agricultural practices, which are distinct from those in other parts of China. For example, Heilongjiang’s climate and black earth (one of the four major black earth regions in the world) make it one of the largest grain-producing areas; however, it also has specific challenges, such as shorter growing seasons and different pest management needs. Given these characteristics, our sampling strategy might have been influenced by local conditions. This may limit the generalizability of our findings in other regions. Future studies should consider these regional differences and expand their sampling to include a more diverse range of agricultural settings to enhance the generalizability of the findings. Furthermore, comparative studies across different regions with varying climatic and agricultural conditions would provide valuable insights into our findings. Secondly, our study targeted the largest developing country, China. However, variations in agricultural production methodologies across different regions suggest that the findings may not be universally applicable. Future research should consider diverse international contexts, including developed nations like the United States and European countries, as well as underdeveloped regions. Investigating the relationship between digital human capital and farmers’ willingness to engage in green production within these varied national settings would significantly enrich the theoretical and practical insights available on this topic.

## 6. Conclusions

This study selects farmers from China’s largest major grain-producing area as its sample, and employs the Probit model and a moderated mediating method to examine the impact of DHC on GPW. The findings indicate that DHC promotes GPW through three dimensions, namely DBC, DCC, and DFPC. Notably, the relationship between these two variables is mediated through FOL rather than IOL effects. Furthermore, the study identifies SN as an indispensable pillar in the promotion of GPW through DHC. Heterogeneity analysis reveals that the effect of DHC on GPW is more pronounced among middle-aged farmers as well as small-scale farmers. Governments in developing countries should speed up the improvement of farmers’ digital human capital level, focusing on expanding access to online for learning green production technologies and promoting social networks to support effective small-scale farmers and middle-aged farmers. By improving the level of digital human capital, it not only comprehensively improves farmers’ willingness to engage in green production but also strengthens the sustainability of agricultural development.

## Figures and Tables

**Table 1:** Weighting measures of online learning.

First Level	Second Level	Third Level	Weights
Online learning	Formal online learning	Online agricultural production knowledge learning frequency organized by the village	0.1787
		Online training frequency of agricultural technology extension personnel	0.1499
		The use of relevant APP about agricultural production recommended by the village leaders	0.1786
	Informal online learning	Learn the relevant content of agricultural green production independently through short video platform	0.1630
		Discuss the experience of agricultural green production with non-professionals online	0.1641
		Use the official account of agricultural WeChat (or on the webpage) to solve the latest agricultural policies and green production information	0.1657

**Table 2:** Weighting measures for online social networks.

First Level	Second Level	Third Level	Weights
Social networks	Network breadth	The total income and expenditure of human relations	0.4517
	Connection strength	Frequency of interaction with relatives, friends, and neighbors	0.2944
	Network’s upward sociability	Frequency of communication with village leaders (and public officials above the village level)	0.2539

**Table 3:** Descriptive statistics.

Variable	Variable Name	Variable Definition	Min	Max	SD
GPW	Green production willingness	Are you willing to make a green production? Yes = 1, No = 0	0	1	0.4677
DHC	Digital human capital	The result as calculated above.	1.4946	4.1991	0.5013
ONL	Online learning	Formal online learning	1	5	0.9923
		Informal online learning	1	5	1.2188
SN	Social network	The result as calculated above.	1	5	0.6067
GEN	Gender	Male = 1, Female = 0	0	1	0.3975
AGE	Age	Actual age (years)	25	65	7.8587
EDU	Education level	Primary school and below = 1, Junior middle school = 2, Senior middle school = 3, College degree and above = 4	1	4	0.9333
PI	Political identity	Are you a village cadre or not? Yes = 1, No = 0	0	1	0.3144
WORK	Part-time employment situation	Have you worked outside your home? Yes = 1, No = 0	0	1	0.4707
AGRI	Agricultural production years	Actual farming years	3	50	10.7910
LABOR	Agricultural labor quantity	Number of households engaged in agricultural production	1	6	0.6543
COO	Participating in the cooperative association	Are you involved in the cooperative association? Yes = 1, No = 0	0	1	0.4660
DIST	Distance to the township	Distance from the nearest town (km)	0	20	4.3022
INCOME	Planting income	Planting income of last year (ten thousand yuan)	0.2	30	6.5400
AREA	Area of cultivated land	Planting area of grain (hm^2^)	0.3334	66.6667	8.9727
LAND	Cultivated land quality	Horrible = 1, Bad = 2, Ordinary = 3, Good = 4, Great = 5	1	5	1.0503
DISA	Natural disasters	Did you suffer from natural disasters last year? Yes = 1, No = 0	0	1	0.4313
INS	Agricultural insurance	Have you purchased agricultural insurance? Yes = 1, No = 0	0	1	0.3604

**Table 4:** Results of the impact of digital Human Capital on farmers’ willingness to engage in green production.

Variables	GPW			
	(1)	(2)	(3)	(4)
	Coefficient	Marginal Effect	Coefficient	Marginal Effect
DHC	0.3862 ***	0.1185 ***		
	(0.0729)	(0.0214)		
DBC			0.1241 ***	0.0379 ***
			(0.0473)	(0.0143)
DCC			0.1165 **	0.0356 **
			(0.0470)	(0.0143)
DCCC			0.0443	0.0135
			(0.0432)	(0.0132)
DFPC			0.0998 **	0.0305 **
			(0.0488)	(0.0148)
GEN	−0.0911	−0.0280	−0.0901	−0.0275
	(0.1633)	(0.0501)	(0.1640)	(0.0502)
AGE	−0.0164	−0.0050	−0.0061	−0.0019
	(0.0642)	(0.0197)	(0.0633)	(0.0194)
EDU	0.0346 ***	0.0106 ***	0.0337 ***	0.0103 ***
	(0.0056)	(0.0016)	(0.0056)	(0.0016)
PI	−0.0373	−0.0114	−0.0307	−0.0094
	(0.1630)	(0.0500)	(0.1632)	(0.0499)
WORK	0.0276	0.0085	0.0289	0.0088
	(0.1034)	(0.0317)	(0.1038)	(0.0317)
AGRI	0.0104 **	0.0032 **	0.0105 **	0.0032 **
	(0.0042)	(0.0013)	(0.0042)	(0.0013)
LABOR	−0.1204 *	−0.0370 *	−0.1269 *	−0.0388 *
	(0.0717)	(0.0220)	(0.0716)	(0.0218)
COO	−0.1244	−0.0382	−0.1333	−0.0408
	(0.1042)	(0.0319)	(0.1053)	(0.0321)
DIST	−0.0225	−0.0069	−0.0240	−0.0073
	(0.0190)	(0.0058)	(0.0191)	(0.0058)
INCOME	0.0077	0.0024	0.0095	0.0029
	(0.0099)	(0.0030)	(0.0099)	(0.0030)
AREA	0.0194	0.0060	0.0139	0.0042
	(0.0610)	(0.0187)	(0.0614)	(0.0188)
LAND	0.1939 ***	0.0595 ***	0.1959 ***	0.0599 ***
	(0.0429)	(0.0128)	(0.0430)	(0.0128)
DISA	0.1108	0.0340	0.1128	0.0345
	(0.1240)	(0.0380)	(0.1250)	(0.0382)
INS	0.0583	0.0179	0.0566	0.0173
	(0.1691)	(0.0519)	(0.1693)	(0.0518)
Region FE	Yes		Yes	
_cons	−3.2266 ***		−3.1299 ***	
	(0.5599)		(0.5568)	
N	854		854	
Pseudo R^2^	0.1152		0.1187	

Note: ***, **, and * indicate significance at the 1% (0.01), 5% (0.05), and 10% (0.1) levels.

**Table 5:** Results of the endogeneity.

Variables	DHC	GPW
	(1)	(2)
Instrumental variable	0.3934 ***	
	(0.1205)	
DHC		0.4557 **
		(0.2295)
F test	18.08 ***	
Control variables	Yes	Yes
Region FE	Yes	Yes
_cons	2.6307 ***	−1.1879 **
	(0.2576)	(0.5726)
N	854	854
R^2^	0.2342	0.0782

Note: *** and ** indicate significance at the 1% (0.01) and 5% (0.05) levels. Due to space limitations, the detailed results of the control variables are not provided. However, all control variables have been included and controlled for in the analysis.

**Table 6:** Results of robustness test.

Variables	GPW					
	(1)	(2)	(3)	(4)	(5)	(6)
	Coefficient	Marginal Effect	Coefficient	Marginal Effect	Coefficient	Marginal Effect
DHC	0.6517 ***	0.1183 ***	0.3589 ***	0.1097 ***	0.3685 ***	0.1126 ***
	(0.1257)	(0.0216)	(0.0630)	(0.0184)	(0.0759)	(0.0224)
Control variables	Yes		Yes		Yes	
Region FE	Yes		Yes		Yes	
_cons	−5.4129 ***		−3.0853 ***		−3.0351 ***	
	(0.9631)		(0.5491)		(0.5694)	
N	854		854		854	
Pseudo R^2^	0.1154		0.1185		0.1085	

Note: *** indicates significance at the 1% (0.01) level. Due to space limitations, the detailed results of the control variables are not provided. However, all control variables have been included and controlled for in the analysis.

**Table 7:** Results of the mediating role of online learning.

Variables	GPW					
	(1)	(2)	(3)	(4)	(5)	(6)
DHC	0.1229 ***			0.0994 ***	0.0894 ***	0.0757 ***
	(0.0237)			(0.0260)	(0.0262)	(0.0276)
FOL		0.0529 ***		0.0314 **		0.0256 *
		(0.0131)		(0.0142)		(0.0145)
IOL			0.0698 ***		0.0408 **	0.0340 **
			(0.0152)		(0.0167)	(0.0171)
Control Variables	Yes	Yes	Yes	Yes	Yes	Yes
Region FE	Yes	Yes	Yes	Yes	Yes	Yes
_cons	−0.4269 ***	−0.2847 *	−0.2872 *	−0.4604 ***	−0.4367 ***	−0.4625 ***
	(0.1635)	(0.1590)	(0.1582)	(0.1626)	(0.1625)	(0.1618)
N	854	854	854	854	854	854
R^2^	0.1380	0.1683	0.1943	0.1750	0.2092	0.2350

Note: ***, ** and, * indicate significance at the 1% (0.01), 5% (0.05), and 10% (0.1) levels. Due to space limitations, the detailed results of the control variables are not provided. However, all control variables have been included and controlled for in the analysis.

**Table 8:** Results of the moderating role of online social networks.

Variables	Online Learning					
	FOL			IOL		
	(1)	(2)	(3)	(4)	(5)	(6)
DHC	0.3938 ***	0.3945 ***	0.3952 ***	0.7261 ***	0.7270 ***	0.7283 ***
	(0.0488)	(0.0488)	(0.0489)	(0.0567)	(0.0566)	(0.0564)
SN		−0.0284	−0.0275		−0.0371	−0.0355
		(0.0543)	(0.0543)		(0.0279)	(0.0278)
DHC × SN			0.0550 *			0.1023 ***
			(0.0332)			(0.0385)
Control variables	Yes	Yes	Yes	Yes	Yes	Yes
_cons	1.3189 ***	1.3389 ***	1.3400 ***	1.9488 ***	1.9750 ***	1.9770 ***
	(0.3306)	(0.3309)	(0.3306)	(0.3836)	(0.3840)	(0.3827)
N	854	854	854	854	854	854
R^2^	0.1328	0.1342	0.1368	0.2260	0.2275	0.2336

Note: *** and * indicate significance at the 1% (0.01) and 10% (0.1) levels. Due to space limitations, the detailed results of the control variables are not provided. However, all control variables have been included and controlled for in the analysis.

**Table 9:** Results of conditional indirect relationships.

	β	SE	95% Boot LLCI	95% Boot ULCI
FOL				
Low-SN (−1SD)	0.3238	0.0648	0.1968	0.4509
Average	0.3952	0.0488	0.2996	0.4907
High-SN (+1SD)	0.4665	0.0654	0.3384	0.5946
IOL				
Low-SN (−1SD)	0.5956	0.0750	0.4486	0.7427
Average	0.7283	0.0564	0.6177	0.8389
High-SN (+1SD)	0.8610	0.0757	0.7127	1.0093

Note: The conditional indirect effect test is based on 20,000 bootstrap resamples.

**Table 10:** Heterogeneity regression results of age.

Variables	GPW					
	Young		Middle-Aged		Elderly	
	(1)	(2)	(3)	(4)	(5)	(6)
	Coefficient	Marginal Effect	Coefficient	Marginal Effect	Coefficient	Marginal Effect
DHC	0.3046 ***	0.0926 ***	0.5602 ***	0.1135 ***	0.4768 ***	0.1097 ***
	(0.0847)	(0.0252)	(0.1594)	(0.0319)	(0.1282)	(0.0295)
Control variable	Yes		Yes		Yes	
Regional FE	Yes		Yes		Yes	
_cons	−3.6817 ***		−5.8295 ***		−5.4228 ***	
	(1.0606)		(1.7128)		(1.6646)	
N	198		585		71	
Pseudo R^2^	0.0716		0.1432		0.1765	

Note: *** indicates significance at the 1% (0.01) level. Due to space limitations, the detailed results of the control variables are not provided. However, all control variables have been included and controlled for in the analysis.

**Table 11:** Heterogeneity regression results of farmland scale.

Variables	GPW			
	Small-Scale		Large-Scale	
	(1)	(2)	(3)	(4)
	Coefficient	Marginal Effect	Coefficient	Marginal Effect
DHC	0.4560 ***	0.1382 ***	0.3378 ***	0.1017 ***
	(0.1018)	(0.0292)	(0.1090)	(0.0318)
Control variable	Yes		Yes	
Regional FE	Yes		Yes	
_cons	−4.5929 ***		−2.5294 ***	
	(1.1095)		(0.8611)	
N	461		393	
Pseudo R^2^	0.1304		0.1275	

Note: *** indicates significance at the 1% (0.01) level. Due to space limitations, the detailed results of the control variables are not provided. However, all control variables have been included and controlled for in the analysis.

## Data Availability

The data presented in this study are available upon request from the first authors.

## Abbreviations

GPW	Green production willingness
DHC	Digital human capital
DBC	digital basic capability
DCC	digital communication capability
DCCC	digital content creation capability
DFPC	digital financial participation capability
ONL	Online learning
FOL	Formal online learning
IOL	Informal online learning
SN	Social network

## Appendix A

**Table A1:** Measurement of Digital Human Capital.

Items	Indicator	Description of Indicator	Option
Digital basic capability	Physical operation of digital devices	I am able to use the general functions of a smartphone, such as making calls.	Strongly Disagree = 1; Disagree = 2; Neutral = 3; Agree = 4; Strongly Agree = 5
	Software operation of digital devices	I can skillfully use apps on my smartphone and computer.	
	Browsing and searching for digital content	I can use a smartphone (computer) to search for and navigate through information.	
	Evaluation of digital content	I can understand information posted on the internet via smartphones.	
	Management of digital content	I can sift through a large volume of information to extract the main points I need.	
Digital communication capability	Digital communication	I frequently use WeChat and other apps to chat with people.	
	Digital sharing	The village officials post announcements in chat groups like WeChat and QQ.	
	Digital participation	I can share my opinions on apps, such as commenting in group chats or on Moments.	
	Digital expression	I respect different viewpoints and positions expressed online.	
	Digital norms	I am able to regulate my behavior and speech online proactively.	
Digital content creation capability	Recording of digital content	I often use my smartphone to take photos and videos to document my daily life.	
	Learning digital content	I frequently learn how to use digital devices to enhance my digital skills (including online shopping, video posting, etc.).	
	Integrating and summarizing digital content	I have experience mimicking online videos to create and post my own content.	
	Understanding of digital copyright	I am aware that information posted online is subject to copyright laws (it cannot be used without permission).	
	Adhering to copyright and license restrictions	When posting content online, I am mindful of copyright issues (for example, not allowing others to misuse photos posted on social media).	
Digital financial participation capability	Level of engagement in digital finance	I use online services for paying taxes, utility bills (such as phone and electricity), booking tickets, and more.	
	Digital consumption	I often use digital payment methods (such as WeChat Pay, Apple Pay, etc.) when shopping (including online shopping).	
	Digital investment	I frequently buy financial management or investment products online, such as Yu Ebao and Licaitong.	
	Digital lending	I frequently make purchases using online lending services such as Ant Flower and Jingdong White Bar.	
	Digital insurance	I frequently buy various types of insurance online, including property, personal, health, and auto insurance.	
